# Remodeling of the Immune Response With Aging: Immunosenescence and Its Potential Impact on COVID-19 Immune Response

**DOI:** 10.3389/fimmu.2020.01748

**Published:** 2020-08-07

**Authors:** Lucas Leite Cunha, Sandro Felix Perazzio, Jamil Azzi, Paolo Cravedi, Leonardo Vidal Riella

**Affiliations:** ^1^Department of Medicine, Escola Paulista de Medicina, Federal University of São Paulo, São Paulo, Brazil; ^2^Division of Rheumatology, Escola Paulista de Medicina, Federal University of São Paulo, São Paulo, Brazil; ^3^Schuster Transplantation Research Center, Brigham and Women's Hospital, Harvard Medical School, Boston, MA, United States; ^4^Renal Division, Department of Medicine, Icahn School of Medicine at Mount Sinai, New York, NY, United States; ^5^Division of Nephrology, Massachusetts General Hospital, Harvard Medical School, Boston, MA, United States; ^6^Department of Surgery, Center for Transplantation Sciences, Massachusetts General Hospital, Boston, MA, United States

**Keywords:** immunosenescence, inflammaging, SARS-CoV-2, COVID-19, immunopathogenesis

## Abstract

Elderly individuals are the most susceptible to an aggressive form of coronavirus disease (COVID-19), caused by SARS-CoV-2. The remodeling of immune response that is observed among the elderly could explain, at least in part, the age gradient in lethality of COVID-19. In this review, we will discuss the phenomenon of immunosenescence, which entails changes that occur in both innate and adaptive immunity with aging. Furthermore, we will discuss inflamm-aging, a low-grade inflammatory state triggered by continuous antigenic stimulation, which may ultimately increase all-cause mortality. In general, the elderly are less capable of responding to neo-antigens, because of lower naïve T cell frequency. Furthermore, they have an expansion of memory T cells with a shrinkage of the T cell diversity repertoire. When infected by SARS-CoV-2, young people present with a milder disease as they frequently clear the virus through an efficient adaptive immune response. Indeed, antibody-secreting cells and follicular helper T cells are thought to be effectively activated in young patients that present a favorable prognosis. In contrast, the elderly are more prone to an uncontrolled activation of innate immune response that leads to cytokine release syndrome and tissue damage. The failure to trigger an effective adaptive immune response in combination with a higher pro-inflammatory tonus may explain why the elderly do not appropriately control viral replication and the potential clinical consequences triggered by a cytokine storm, endothelial injury, and disseminated organ injury. Enhancing the efficacy of the adaptive immune response may be an important issue both for infection resolution as well as for the appropriate generation of immunity upon vaccination, while inhibiting inflamm-aging will likely emerge as a potential complementary therapeutic approach in the management of patients with severe COVID-19.

## Introduction

In December 2019, a novel coronavirus, severe acute respiratory syndrome coronavirus-2 (SARS-CoV-2), was discovered as the causative agent of an outbreak of viral lower-respiratory tract infections centered in Wuhan (China) (1). Since then, SARS-CoV-2 has caused a widespread outbreak of severe acute respiratory syndrome throughout China, with exported cases occurring in other continents, including the United States, in a worldwide pandemic (1). Interestingly, a strong age gradient in the risk of death was observed among patients with coronavirus disease (COVID-19) (2). In this scenario, the remodeling of immune response that is observed among the elderly could be a possible explanation for the higher lethality of COVID-19 noted on this population.

The immune response is dynamically remodeled with aging, a phenomenon denominated as immunosenescence. This phenomenon increases susceptibility to a myriad of clinical conditions such as infections, autoimmune disorders, and malignancies. Recent data had shed light on the physiological aspects of immunosenescence, which is now considered an immune adaptation to the aged microenvironment rather than merely a collapse of the system (3).

Both the innate and adaptive immunity is affected by aging. Some individuals experience a sustained innate immune system activation, inducing proinflammatory cytokines secretion and innate immune cells' recruitment (4). Innate immunity hyperactivation may be detrimental and impair global functionality, causing a clinical phenotype known as frailty syndrome. Frailty syndrome is defined as a state of cumulative decline in several physiological systems with a disproportionate vulnerability to stressor events (5). Frailty syndrome prevalence increases with age, it is multifactorial in etiology, and the physical component of frailty can be objectively assessed by the Fried Frailty Score (Phenotype Score) and the Frailty Index (Deficit Accumulation Index) (6).

Likewise, adaptive immunity remarkably changes as age increases, which can be summarized into two main topics: (1) bone marrow reorganization and hematopoietic stem cell pool differentiation into myeloid lineage, outnumbering lymphoid compartment; and (2) physiological thymic involution, compromising naïve T cells generation. The sum of these two factors can help explain the prior known impairment of the regenerative capacity of lymphocytes compared to myeloid-derived cells in the elderly (7).

Infectious diseases are more prevalent among the elderly. When compared to younger counterparts, the elderly more frequently present with respiratory and urinary tract infections, and those patients usually have a worse prognosis (8, 9). It is possible that the impaired barrier function of mucosae and diminished adaptive immune response (both cellular and humoral) are the reasons for the increased susceptibility to infectious microorganisms among the elderly (10). In addition, the natural killer (NK) cell senescence may affect the homeostasis of the immune system in the elderly, leading to an increased risk of cancer and additional risk of viral infections (11). Lastly, age-related cell dysfunctions leading to an exhausted phenotype are also an important characteristic of the immune system remodeling with aging, which might accelerate tissue damage and disable modulatory mechanisms (12). Herein, we review the state of the art research on senescence-induced immune dysregulation, focusing on innate and adaptive cell functional analysis and its potential impact in viral immune responses, such as in COVID-19.

## Physiology of Immunosenescence and Inflamm-Aging

Currently, the concept of immunosenescence refers to a comprehensive remodeling of the immune system and its microenvironment, involving both innate and adaptive compartments that occur with aging (13, 14). Many physiological phenomena have been proposed to explain the immune response remodeling over time, including chronic exposure to antigens, impaired telomerase activity, mitochondrial dysfunction, defective autophagy, endoplasmic reticulum stress, defective ubiquitin/proteasome system, and age-related changes in the composition of gut microbiota (15–18). Probably, a melting pot of diverse factors differently contributes to the final phenotype of the adapted and experienced immune system, named immunosenescence.

Aging of the immune system is characterized by an imbalance between stimulatory and regulatory mediators, such as cytokines and acute phase reactants, toward a sub-clinical chronic proinflammatory state called inflamm-aging. Inflamm-aging is thought to be caused by a low-grade inflammation secondary to continuous antigenic stimulation (19), whose source may be exogenous, like a pathogenic microorganism infection (20, 21), or endogenous (15–18), like post-translational-modified macromolecules (15). Population studies incorporate the notion that the immune response depends on environmental exposure and how it interacts with endogenous variables. In fact, diet, exercise, xenobiotic exposure, and other environmental factors may epigenetically affect the metabolic health of immune cells (22). Lifestyle factors, such as exercise and favorable dietary habits, positively affect the immune system (22), while poor nutrition and reduced muscle mass may predispose an individual to a proinflammatory condition (23).

### Innate Immune Response and Inflamm-Aging

Age-related remodeling of innate immunity modifies the homeostasis of NK cells, neutrophils, and monocytes/macrophages (24). NK cells from the elderly exhibit impaired perforin release upon stimulation and granule exocytosis (25, 26). It reduces the elimination of senescent cells, which, in turn, promotes senescent cell accumulation in aged tissue. Moreover, aging reduces the frequency of circulating NK p46^+^ cells, a modulatory cell subset involved in the resolution of inflammation and elimination of effector cells (27, 28).

Neutrophils and macrophages are classically classified as part of innate immunity and possibly comprise the most important effector cells against bacterial infections. It is thought that age is accompanied by a decline in production and secretion of most chemokines, including those responsible for neutrophil and monocyte chemoattraction (29). The absolute number of neutrophils seems to be maintained while the number of monocytes increase with age (30, 31). However, the function of these cells may be impaired among the elderly (32). The final consequence is that the delayed resolution of inflammation may be associated with age-related remodeling of neutrophils and macrophages (29).

In addition to their phagocytosis' capabilities, neutrophils are capable of releasing a mesh-like structure under specific circumstances, called neutrophil extracellular traps (NET), in an attempt to physically delimitate the pathogenic agent, mainly microorganisms, and facilitate its contact with microbicidal peptides and enzymes (33). NET is composed of a decondensed chromatin meshwork imbedded with granule proteins with anti-microbial properties. NET may also work as a physical path for immune cell migration to the inflammatory site (34). Neutrophil function is impaired in both animal models and humans with aging. Hazeldine et al. (35) observed that older adults have less IL-8 production, LPS-induced NET release, and cell migration compared to younger counterparts, probably secondary to an impaired signal transduction. Microbicidal killing, phagocytic activity (36), and degranulation capacity (37) of neutrophils are also reduced in the elderly. In addition, the same group investigated the migration pattern of neutrophils obtained from older compared to young adults. They observed that neutrophils from older subjects migrated with less accuracy than those from younger subjects. By inaccurately meandering among healthy tissues, neutrophils from the elderly inadvertently release more neutrophil proteinase that may contribute to tissue damage and systemic inflammation.

Reactive oxygen species (ROS) are free radicals produced after oxidative bursts in phagosomes, which are pivotal for the microbicidal function of phagocytes (38). In fact, ROS do not just directly contribute to the bacterial clearance, but additionally can trigger NET formation. The free radical ROS production by neutrophils in older adults is decreased (39, 40). Interestingly, polymorphonuclear leucocytes from the elderly are less capable of modulating the triggering receptor expressed on myeloid cell-1 (TREM-1)-induced oxidative bursts, suggesting that TREM-1 signal transduction altered with aging may be one of the mediators of the decrease in microbicidal potential of innate immune cells in older adults (41).

Animal models of premature immunosenescence have also shed some light into age-related remodeling of the immune system. Guayerbas et al. (42) described a mouse model of premature immunosenescence based on the demonstration of early decline of immune parameters and behavioral tests in Swiss outbred mice. Mouse model-derived peritoneal leukocytes exhibited reduced proliferative response, impaired NK activity, and increased *in vitro* TNF-alpha production compared to control mice (42). In addition, mouse model-derived macrophages of premature models were less functional with a striking loss of microbicidal activity (43).

The mice model of premature immunosenescence was refined and new models were developed as well (44, 45). Apparently, the key phenomenon are the oxidative and inflammatory stresses, which, not without reason, are associated with several non-communicable chronic diseases prevalent among the elderly (44, 46). In fact, spleen and thymus cells from prematurely immunosenescent mice models have decreased antioxidant defenses and significantly increased oxidants and pro-inflammatory cytokines production (44–46). Interestingly, the antioxidant vs. oxidant imbalance observed in prematurely immunosenescent mice was similar to the one observed in old wild-type animals (44, 47). Hence, lab tests determining the oxidative burst profile of phagocytes (e.g., nitro blue tetrazolium test, dihydrorhodamine oxidation, ${\text{O}}_{2}^{-}$ and H_2_${\text{O}}_{2}^{-}$ production by chemoluminescence, etc.) may be useful for assessing inflamm-aging features (4).

The state of chronic inflammation has to be counter-balanced by anti-inflammatory molecules (48). When not under control, the low-grade inflammation loses its defense role and turns into a damaging state to the whole organism (49). The practical consequence is that inflamm-aging is deleterious to human health, predicts frailty, and is associated with higher mortality rates (50–52).

### Remodeling of the Adaptive Immune System With Aging

Remodeling of the adaptive immune response also occurs with aging. Thymic involution and hematopoietic stem cell insufficiency play important roles in immunosenescence of adaptive immunity (53). In general, elderly individuals are less able to respond to neo-antigens, due to the reduction of new thymus-emergent T cells, though homeostatic proliferation can partially sustain the richness of the TCR repertoire (54, 55). Moreover, peripheral T cells usually present a reduced absolute number in aged individuals with an inverted CD4:CD8 ratio and expansion of terminally differentiated effector memory T cells (56, 57), associated with impaired proliferation ability, telomerase activity, and intracellular signaling (58, 59). Furthermore, most adult regulatory T lymphocytes are a terminally differentiated highly suppressive apoptosis-prone population with a limited capacity for self-renewal (60). This finding might explain, at least in part, the occurrence of age-related autoimmune conditions. In addition, the imbalance between innate and adaptive immunity may disturb the fine regulation of the effector immune response, leading to a severe acute pro-inflammatory state that may lead to organ rejection in transplanted patients (61, 62).

While naïve T and B cells become dysfunctional with aging, memory T and B cells' function is relatively maintained (63–65). In fact, naïve T lymphocytes obtained from the elderly present impaired cell binding of the immune synapse (66), reduced signal transduction (67), dysregulation of cytoskeletal function (68), defective protein glycosylation and activation (69), and insufficient IL-2 production (70).

**Figure 1:**
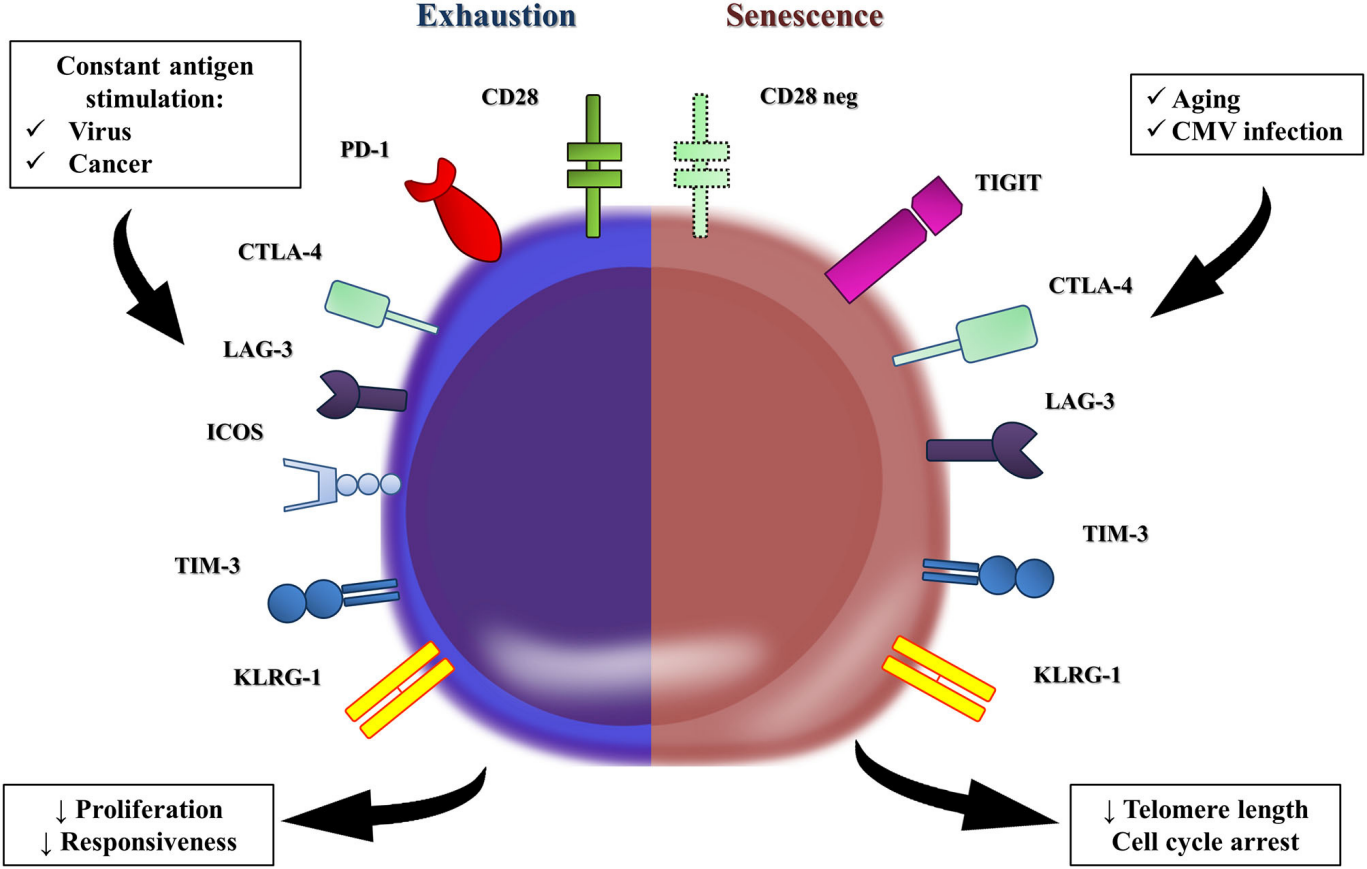
T cell exhaustion vs. T cell senescence. In conceptual terms, the T-cell dysfunction observed in the elderly is different to the one reported as T-cell exhaustion. Persistent viral and cancer stimulation leads to the remodeling of many T cells, which upregulate the expression of co-inhibitory receptors (e.g., PD-1, CTLA-4, LAG-3, ICOS, Tim-3, and KLRG-1), all of them hallmarks of T cell exhaustion. The co-inhibitory receptors downregulate the TCR-stimulated intracellular signal, and T-cells become hyporesponsive and develop responsiveness impairment. However, the immunosenescence is marked by similar levels of PD-1 and TIM-3 and tiny elevations of CTLA-4 and LAG-3 in T cells from the elderly compared to those in younger groups. T-cell immunoglobulin and immunoreceptor tyrosine-based inhibitory motif (ITIM) domain (TIGIT) is a co-inhibitory receptor that is expressed on senescent T cells, which exhibited a marked terminal differentiated phenotype. Interestingly, TIGIT-positive T cells from the elderly seems to retain some proliferative capacity, but produced significantly lower amounts of TNF-alpha, IFN-gamma, and IL-2.

Some authors advocate that age-related T-cell dysfunction is different from T cell exhaustion, a state of low cell responsiveness mediated by chronic conditions, such as viral infections and malignancies (Figure 1) (71). Constant antigen stimulation progressively exhausts T cells by gradually upregulating the expression of inhibitory checkpoint receptors (e.g., PD-1, CTLA-4, LAG-3, ICOS, Tim-3, and KLRG-1) on CD4^+^ T cells (72), which, in turn, downmodulate TCR-induced intracellular signaling (73). Interestingly, despite this conceptual difference between immunosenescence and T cell exhaustion, most of those cell exhaustion surface hallmarks are observed on dysfunctional immunosenescent cells, suggesting that these two phenomena share many mechanisms (54).

Exhausted T-cells accumulate over time (67, 74–77). Shimada et al. (74) demonstrated both gene and protein hyper expression of PD-1 and CTLA-4 in cells from old male C57BL/6 mice compared to young controls. Most PD-1^+^ T cells were quiescent and presented an anergic effector memory phenotype with impaired proliferative response to mitogens (74). Similarly, Lee et al. (76) reported the accumulation of Tim-3^+^ murine T cells with impaired proliferative capacity with aging.

Literature discussing T cell exhaustion and immunosenescence in humans is scarce, though. Song et al. (77) described an elevated number of TIGIT^+^ CD8^+^ T cells from old adults, another hallmark of cell exhaustion apparently associated with immunosuppressant features in neoplasm or chronic infection mouse models (78, 79). TIGIT^+^ CD8^+^ T cells from old individuals seem to retain a proliferative capacity, although they impaired TNF-alpha, IFN-gamma, and IL-2 *in vitro* production and increased susceptibility to apoptosis (77). Therefore, we hypothesize that evaluation of the proliferative response to mitogens and *in vitro* cytokine production may be indirect ways to assess age-related remodeling of the immune system.

In regards to B cell compartment, vaccine trials suggest that B cell repertoire abridge over time, foremost observed in frail patients (80, 81). In addition, B cells from the elderly present both impaired antibody production and class switch recombination (82). Class switch recombination and immunoglobulin somatic hypermutation are crucial for humoral immune response and occur in mature B cells mediated by activation-induced cytidine deaminase, amongst other mediators (82, 83). Similarly, activated B cells from old mice have less activation-induced cytidine deaminase expression and reduction of class switched antibodies (84, 85). Interestingly, *in vivo* activated CD4^+^ T cells from old-aged individuals showed increased dual-specific phosphatase 4 (DUSP4) transcription, which, in turn, negatively correlated with antigen-specific B cells' expansion. Silencing of DUSP4 restored CD4^+^ T cell-induced B-cell differentiation, suggesting that B cell dysfunction observed with aging is T cell-dependent. Table 1 summarizes the main physiologic modifications of the immune system in the elderly.

**Table 1:** Summary of the age-related physiologic modifications of the immune system.

**Cell**	**Immune response**	**Aging functional impairment**	**Clinical impact**	**References**
NK	Innate	• ↓ Perforin degranulation	• Wound healing • Susceptibility to infection • Susceptibility to cancer	(26)
Neutrophil	Innate	• ↓ Phagocytosis • ↓ ROS production • ↓ Intracellular killing • ↓ NET • ↓ Migration	• Wound healing • Susceptibility to infection	(35, 86, 87)
Basophil	Innate	• Delayed degranulation	• Susceptibility to helminth infection • Decrease in allergy parameters	(88–90)
Eosinophil	Innate	• Delayed degranulation • ↓ Superoxide production	• Susceptibility to helminth infection • Decrease in allergy parameters	(88, 91)
T cell	Adaptive	• ↓ Repertoire • Relative decrease of naïve T cells • Relative increase of memory T cells	• Impaired response to vaccination • Susceptibility to infection	(54–60)
B cell	Adaptive	• ↓ Repertoire of antibodies	• Impaired response to vaccination • Susceptibility to infection	(80–82, 84, 85)
Dendritic cells	Adaptive	• ↓ Antigen presentation • ↓ Tolerant response	• Susceptibility to skin and mucosal infection • Susceptibility to autoimmune disorder • Impaired response to vaccination • Transplant rejection	(31, 92–94)

*Arrow means diminished*.

## Immune Response, Immunosenescence, and COVID-19

Coronaviruses are a large family of viruses that cause upper and lower-respiratory tract illnesses in humans. SARS-CoV-2 is transmitted predominantly via respiratory droplets. Clinically, patients frequently present with fever, cough, myalgia, and fatigue (95). In a subset of patients, mainly elderly individuals, SARS-CoV-2 was shown to lead to bilateral pulmonary diffuse alveolar damage that may progress to acute respiratory distress syndrome (96, 97). Following the pulmonary phase, patients with poor outcome frequently evolve a life-threatening cytokine storm syndrome, characterized by bursts of pro-inflammatory cytokines and chemokines in the serum (96, 98). The uncontrolled systemic inflammation causes endothelial injury and activation of coagulation cascade. The consequence is an explosive process of disseminated intravascular coagulation and consumption of coagulation factors that leads to organ damage and death.

### Innate Immune Response and COVID-19

The innate immune response is the first level of response in the detection and clearance of a viral infection. In SARS-CoV-2, the spike protein (S) mediates the attachment, fusion, and entry of the virus in human cells (99). The protein S strongly binds to angiotensin-converting enzyme 2 receptor leading to the attachment of the virus to the host cell (99). The successful entry needs the priming of the S protein by TMPRSS2, a human cellular serine protease (100). Once in the host cell, SARS-CoV-2 can be detected by macrophages, which orchestrate the production of a pro-inflammatory microenvironment that inhibits viral replication, stimulates adaptive immunity, and recruits other immune cells to the site of infection.

Macrophages from elderly lungs may have a more pronounced production of IL-6 and other pro-inflammatory cytokines in response to stimuli (101). It is possible that IL-6 has a critical role in the immune response of the elderly that mounts against SARS-CoV-2 (102). IL-6 helps the differentiation of Th17 lymphocytes, but inhibits the production of Interferon-γ, which is necessary for the activation of CD8+ cells (102). In addition, IL-6 contributes to a pro-inflammatory microenvironment at the lung that impacts the integrity of the air-blood barrier (103). Patients with severe COVID-19 have a higher IL-6/Interferon-γ ratio than those who present with a moderate disease, which could be related to the cytokine storm leading to lung injury (104–107). Indeed, patients with severe COVID-19 frequently have lower absolute numbers of Interferon-γ producing CD4+ T cells compared to patients with moderate disease (108). Then, when patients with COVID-19 enter the immune dysregulation phase, the increase in IL-6 leads to a relative immunoparalysis that may impair the clearance of SARS-Cov-2 (98). Elderly patients with COVID-19 often present with a severe dysregulation of pro-inflammatory cytokines, such as IL-6 and IL-1 β, which may result in worse outcome (105). Drugs that uncouple IL-1β/IL-1R signaling (anakinra) or IL-6/IL-6R signaling (tocilizumab) may have an immunomodulatory potential and are hypothesized to attenuate the dysfunctional immune response during the hyperinflammatory phase of COVID-19 (98, 109). In fact, some reports suggest that infusion of anakinra (109, 110) and tocilizumab (111) may improve the disease course in patients with severe COVID-19 presentation.

Neutrophils have traditionally been considered the primary immune cells active in the defense against bacterial infections. More recently, neutrophils' role in viral infection has emerged based on observations of its correlation with viral infection severity and neutrophils' biological ability to recognize viruses (via viral PAMPs) and respond to them with specific effector functions (112). Patients with severe COVID-19 more frequently present with a high neutrophil-to-lymphocyte ratio (113), in part driven by the relative lymphopenia or lymphocyte exhaustion. In addition, patients with severe COVID-19 are more susceptible to a greater burst of systemic inflammation and secondary bacterial infection that can lead to the increment of neutrophils. It is unclear if changes in neutrophils are only a reflection of the overall immune activation in COVID-19 or if they play a direct pathogenic role. Lastly, NK cells are less functional in the elderly, and studies have shown that severe COVID-19 patients have further depleted peripheral NK cell counts in comparison with mild cases and healthy controls (114–116). Generally, NK cells are capable of recognizing infected cells and of triggering direct cell toxicity. Further studies are needed to clarify how SARS-CoV2-infected cells interact with NK cells and if any apoptosis or downmodulation occurs and prevents the effective elimination of infected cells.

The airway epithelium is a physical barrier to pathogens (117). The integrity of the air-blood barrier is essential for the maintenance of lung homeostasis and represents an important branch of innate immunity (118). The invasion of the airway epithelial by SARS-CoV-2 may break the barrier integrity, triggering a vicious cycle of inflammation and tissue injury that is more pronounced among the elderly (119). Presumably, the same remodeling process that occurs in the immune system also happens at the lung microenvironment with aging (120). Data from animal models suggest that senescent lungs are more susceptible to settle a pro-inflammatory response when injured (121). In fact, bronchoalveolar lavage obtained from elderly patients with acute respiratory distress syndrome present with higher pro-inflammatory cytokine levels when compared to younger counterparts, suggesting that the lung may represent a small fraction of the inflamm-aging that occurs at the systemic level (122). This local phenomenon may help to explain why elderly patients with COVID-19 are more susceptible to a more severe lung injury that implies loss of lung function and respiratory failure (123).

### Adaptive Immune Response and COVID-19

The initial inflammation in COVID-19 is propitious to the activation and differentiation of CD4+ and CD8+ T cells. The ideal final output is the development of an effective and specific immune response, involving both the production of anti- SARS-CoV-2 antibodies and the deployment of a large number of viral-specific cytotoxic lymphocytes that will ultimately eliminate the virus and achieve clinical recovery. In fact, when compared to severe H7N9 disease, reduced pro-inflammatory cytokines and chemokines were found in COVID-19 patients with good prognosis, reinforcing the idea that adaptive immunity is a key factor for a favorable outcome (124).

Thevarajan et al. (124) described a kinetic of the immune response in a 47-year-old woman with COVID-19 who presented a favorable outcome. They evidenced a persistent increase in antibody-secreting cells, follicular helper T cells, activated CD4+ and CD8+ T cells, and immunoglobulin M (IgM) and IgG antibodies that bound to SARS-CoV-2. The peak of both antibody-secreting cells and follicular helper T cells was markedly higher in the patient compared to healthy controls and both cell subsets were persistently increased during convalescence (day 20). The experience from the SARS epidemic of 2003 showed that convalescent SARS patients present with neutralizing antibodies against S protein (125). The sera stored from convalescent patients from the SARS epidemic of 2003 can cross-neutralize the S protein-mediated SARS-CoV-2 entry in patients with COVID-19 (100). This data raises the possibility that the S protein could be an important antigen to vaccine protocols. In fact, in analogy to the SARS epidemic of 2003, convalescent patients with SARS may present IgG and neutralizing antibodies peaking at 4 months after the disease and detectable up to 2 years afterwards, suggesting that memory B cells can be elicited during coronavirus infection (125).

Cellular immune response may play a critical role in the adaptive immune response in patients with COVID-19. Thevarajan et al. (124) observed the emergence and rapid increase in activated CD8+ T cells at days 7–9 after infection preceded the resolution of symptoms of one young patient with a good prognosis. Conversely, elderly patients and those requiring intensive care unit support presented a dramatically reduced number of CD4+ and CD8+ T cells (126). Lower total amounts of T cells, CD4+, and CD8+ T cells negatively correlated with patient survival (126). Diao et al. (126) noted that T cell absolute counting were negatively correlated to serum IL-6, IL-10, and TNF-α concentration in patients with COVID-19, suggesting that the failure of the adaptive immune response and the increase of pro-inflammatory cytokine may be associated with worse survival. It is also possible that increased IL-6 leads to a reduction in CD4^+^ T cells and NK cells in patients with COVID-19 and immune dysregulation (98). In fact, some pro-inflammatory cytokines, such as IL-6, may block the antiviral immune response by favoring T cells' exhaustion (102). Diao et al. further characterized the exhaustion status of 14 patients with COVID-19. They noted an increasing PD-1 and Tim-3 expression on T cells as patients progressed from prodromal to overtly symptomatic stages (126). Whether this reflects the emergence of exhaustive T cells with a defective capacity to eliminate the virus or a normal evolution of the immune response against the virus remains to be determined. If greater severity of disease is seen in patients with a higher frequency of exhausted T cells, a potential therapeutic approach could be attempted to block those inhibitory receptors, unleashing the T cell response against the virus.

**Figure 2:**
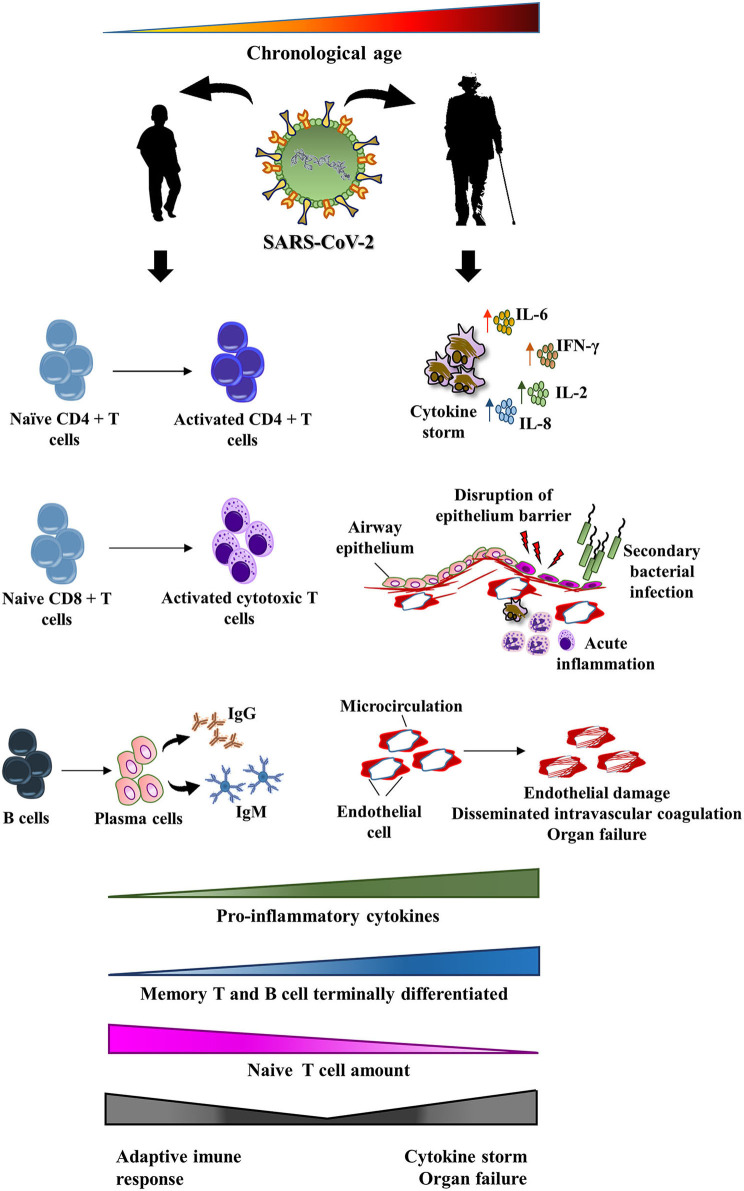
Potential impact of immunosenescence on the pathogenesis of COVID-19. SARS-CoV-2 infection may affect all age ranges, from children to the elderly. Among children, a mild-symptom disease usually occurs. They frequently crush the viral infection through an effective adaptive immune response. However, the remodeling of the immune system that happens with aging may lead to modifications in both adaptive and innate immunity. The final result of these changes may trigger a maladaptive immune response against SARS-CoV-2. In fact, the elderly are an at-risk group to a more aggressive disease that includes cytokine release syndrome, disruption of intrinsic lung defense, secondary bacterial pneumonia, endothelial injury, and end organ damage.

The diminished naïve T cell repository observed among the elderly may dramatically affect the adaptive immune response against SARS-CoV-2, since fewer naïve T cells will be capable of responding to new infections (127, 128). Furthermore, there is also a reduction in the number of regulatory T cells with aging, which help keep the immune system under tighter control (129). Since the elderly frequently present with a remodeled adaptive immune response, they may fail to enhance antibody production. Instead, a pro-inflammatory tone characteristic of inflamm-aging may convert the immune response of patients with COVID-19 in a life-threatening cytokine storm. On the contrary, young patients usually present with an enormous number of naïve T cells that had never encountered a virus. Then, naïve T lymphocytes are rapidly primed and innate immunity does not overwhelm the adaptive immune response. This may explain, at least in part, the favorable prognosis observed among young subjects. Figure 2 shows the possible relationship between immune response in patients with COVID-19 and the remodeling process that takes place in the immune system with aging.

## Conclusion and Future Perspectives

The immune system faces a complex adaptation over time, culminating in functional and phenotyping alterations. The influence of age-related remodeling of the immune system is clinically observed within elderly features (e.g., frailty syndrome) that can be assessed by lab tests. Despite several promising experimental methods, none are clinically validated so far, but certainly shed some light on the pathophysiology of immunosenescence. Novel mechanisms of inflamm-aging may rise in the near future, leading to new potential therapeutic targets for age-related disorders. Different from the chronological age, the “immune age” obtained by population studies may accurately reflect the molecular and cellular changes that occur over time (130). Immunosenescence may explain the lethality amongst the elderly with COVID-19 with a combination of ineffective T cell response, failed antibody production against SARS-CoV-2, and inflamm-aging that terribly collapses the homeostasis, leading to severe organ dysfunction. The biomarkers that are hallmarks of the remodeled immune response have been raised as new potential targets in patients with COVID-19. More studies are warranted to investigate how to help the elderly to elicit a functional adaptive immune response, as well as to diminish the harmful pro-inflammatory state of the disease.

## Author Contributions

LC: conception and design, review of the literature, composition of the manuscript and final approval. SP, JA, and PC: design, critical review of the literature, composition of the manuscript, and final approval. LR: conception and design, selection of notable articles for review, critical review of the literature, composition of the manuscript, clinical, and translational orientation and final approval. All authors contributed to the article and approved the submitted version.

## Conflict of Interest

The authors declare that the research was conducted in the absence of any commercial or financial relationships that could be construed as a potential conflict of interest.

## References

[1] NIH Coronavirus (COVID-19). National Institutes of Health (2020). Available online at: https://www.nih.gov/health-information/coronavirus (accessed April 19, 2020).

[2] Verity R Okell LC Dorigatti I Winskill P Whittaker C Imai N. Estimates of the severity of coronavirus disease 2019: a model-based analysis. Lancet Infect Dis. (2020) S1473–3099:30243–7. 10.1016/S1473-3099(20)30243-7 32240634 PMC7158570

[3] Weng NP. Aging of the immune system: how much can the adaptive immune system adapt? Immunity. (2006) 24:495–9. 10.1016/j.immuni.2006.05.001 16713964 PMC2266981

[4] Fulop T Larbi A Dupuis G Le Page A Frost EH Cohen AA. Immunosenescence and inflamm-aging as two sides of the same coin: friends or foes? Front Immunol. (2017) 8:1960. 10.3389/fimmu.2017.01960 29375577 PMC5767595

[5] Sadighi Akha AA. Aging and the immune system: An overview. J Immunol Methods. (2018) 463:21–6. 10.1016/j.jim.2018.08.005 30114401

[6] Rohrmann S. Epidemiology of Frailty in Older People. Adv Exp Med Biol. (2020) 1216:21–7. 10.1007/978-3-030-33330-0_3 31894543

[7] Kovtonyuk LV Fritsch K Feng X Manz MG Takizawa H. Inflamm-aging of hematopoiesis, hematopoietic stem cells, and the bone marrow microenvironment. Front Immunol. (2016) 7:502. 10.3389/fimmu.2016.00502 27895645 PMC5107568

[8] Lindenauer PK Lagu T Shieh MS Pekow PS Rothberg MB. Association of diagnostic coding with trends in hospitalizations and mortality of patients with pneumonia, 2003-2009. JAMA. (2012) 307:1405–13. 10.1001/jama.2012.384 22474204

[9] Rebelo M Pereira B Lima J Decq-Mota J Vieira JD Costa JN. Predictors of in-hospital mortality in elderly patients with bacteraemia admitted to an Internal Medicine ward. Int Arch Med. (2011) 4:33. 10.1186/1755-7682-4-33 21970460 PMC3206823

[10] Hazeldine J Lord JM. Innate immunesenescence: underlying mechanisms and clinical relevance. Biogerontology. (2015) 16:187–201. 10.1007/s10522-014-9514-3 25009085

[11] Hazeldine J Lord JM. The impact of ageing on natural killer cell function and potential consequences for health in older adults. Ageing Res Rev. (2013) 12:1069–78. 10.1016/j.arr.2013.04.003 23660515 PMC4147963

[12] Aiello A Farzaneh F Candore G Caruso C Davinelli S Gambino CM. Immunosenescence and its hallmarks: how to oppose aging strategically? A review of potential options for therapeutic intervention. Front Immunol. (2019) 10:2247. 10.3389/fimmu.2019.02247 31608061 PMC6773825

[13] Globerson A Effros RB. Ageing of lymphocytes and lymphocytes in the aged. Immunol Today. (2000) 21:515–21. 10.1016/S0167-5699(00)01714-X 11071531

[14] Pawelec G. Immunosenescence: impact in the young as well as the old? Mech Ageing Dev. (1999) 108:1–7. 10366035 10.1016/s0047-6374(99)00010-x

[15] Byun HO Lee YK Kim JM Yoon G. From cell senescence to age-related diseases: differential mechanisms of action of senescence-associated secretory phenotypes. BMB Rep. (2015) 48:549–58. 10.5483/BMBRep.2015.48.10.122 26129674 PMC4911181

[16] Biagi E Candela M Fairweather-Tait S Franceschi C Brigidi P. Aging of the human metaorganism: the microbial counterpart. Age (Dordr). (2012) 34:247–67. 10.1007/s11357-011-9217-5 21347607 PMC3260362

[17] Bauer ME Fuente Mdl. The role of oxidative and inflammatory stress and persistent viral infections in immunosenescence. Mech Ageing Dev. (2016) 158:27–37. 10.1016/j.mad.2016.01.001 26773975

[18] Franceschi C Garagnani P Vitale G Capri M Salvioli S. Inflammaging and ‘Garb-aging’. Trends Endocrinol Metab. (2017) 28:199–212. 10.1016/j.tem.2016.09.005 27789101

[19] Bellon M Nicot C. Telomere dynamics in immune senescence and exhaustion triggered by chronic viral infection. Viruses. (2017) 9:289. 10.3390/v9100289 28981470 PMC5691640

[20] Pita-Lopez ML Gayoso I DelaRosa O Casado JG Alonso C Muñoz-Gomariz E. Effect of ageing on CMV-specific CD8 T cells from CMV seropositive healthy donors. Immun Ageing. (2009) 6:11. 10.1186/1742-4933-6-11 19715573 PMC2741428

[21] Pawelec G Gouttefangeas C. T-cell dysregulation caused by chronic antigenic stress: the role of CMV in immunosenescence? Aging Clin Exp Res. (2006) 18:171–3. 10.1007/BF03327436 16702790

[22] Weyh C Krüger K Strasser B. Physical activity and diet shape the immune system during aging. Nutrients. (2020) 12:622. 10.3390/nu12030622 32121049 PMC7146449

[23] Duggal NA Niemiro G Harridge SDR Simpson RJ Lord JM. Can physical activity ameliorate immunosenescence and thereby reduce age-related multi-morbidity? Nat Rev Immunol. (2019) 19:563–72. 10.1038/s41577-019-0177-9 31175337

[24] Brubaker AL Rendon JL Ramirez L Choudhry MA Kovacs EJ. Reduced neutrophil chemotaxis and infiltration contributes to delayed resolution of cutaneous wound infection with advanced age. J Immunol. (2013) 190:1746–57. 10.4049/jimmunol.1201213 23319733 PMC3563860

[25] Sagiv A Biran A Yon M Simon J Lowe SW Krizhanovsky V. Granule exocytosis mediates immune surveillance of senescent cells. Oncogene. (2013) 32:1971–7. 10.1038/onc.2012.206 22751116 PMC3630483

[26] Hazeldine J Hampson P Lord JM. Reduced release and binding of perforin at the immunological synapse underlies the age-related decline in natural killer cell cytotoxicity. Aging Cell. (2012) 11:751–9. 10.1111/j.1474-9726.2012.00839.x 22642232

[27] Almeida-Oliveira A Smith-Carvalho M Porto LC Cardoso-Oliveira J Ribeiro AoS Falcão RR. Age-related changes in natural killer cell receptors from childhood through old age. Hum Immunol. (2011) 72:319–29. 10.1016/j.humimm.2011.01.009 21262312

[28] Sapey E Greenwood H Walton G Mann E Love A Aaronson N. Phosphoinositide 3-kinase inhibition restores neutrophil accuracy in the elderly: toward targeted treatments for immunosenescence. Blood. (2014) 123:239–48. 10.1182/blood-2013-08-519520 24191150 PMC3888290

[29] Swift ME Burns AL Gray KL DiPietro LA. Age-related alterations in the inflammatory response to dermal injury. J Invest Dermatol. (2001) 117:1027–35. 10.1046/j.0022-202x.2001.01539.x 11710909

[30] Beerman I Bhattacharya D Zandi S Sigvardsson M Weissman IL Bryder D. Functionally distinct hematopoietic stem cells modulate hematopoietic lineage potential during aging by a mechanism of clonal expansion. Proc Natl Acad Sci U S A. (2010) 107:5465–70. 10.1073/pnas.1000834107 20304793 PMC2851806

[31] Della Bella S Bierti L Presicce P Arienti R Valenti M Saresella M. Peripheral blood dendritic cells and monocytes are differently regulated in the elderly. Clin Immunol. (2007) 122:220–8. 10.1016/j.clim.2006.09.012 17101294

[32] Shaw AC Joshi S Greenwood H Panda A Lord JM. Aging of the innate immune system. Curr Opin Immunol. (2010) 22:507–13. 10.1016/j.coi.2010.05.003 20667703 PMC4034446

[33] Brinkmann V Reichard U Goosmann C Fauler B Uhlemann Y Weiss DS. Neutrophil extracellular traps kill bacteria. Science. (2004) 303:1532–5. 10.1126/science.1092385 15001782

[34] Carmona-Rivera C Khaznadar SS Shwin KW Irizarry-Caro JA O'Neil LJ Liu Y. Deficiency of adenosine deaminase 2 triggers adenosine-mediated NETosis and TNF production in patients with DADA2. Blood. (2019) 134:395–406. 10.1182/blood.2018892752 31015188 PMC6659253

[35] Hazeldine J Harris P Chapple IL Grant M Greenwood H Livesey A. Impaired neutrophil extracellular trap formation: a novel defect in the innate immune system of aged individuals. Aging Cell. (2014) 13:690–8. 10.1111/acel.12222 24779584 PMC4326942

[36] Butcher SK Chahal H Nayak L Sinclair A Henriquez NV Sapey E. Senescence in innate immune responses: reduced neutrophil phagocytic capacity and CD16 expression in elderly humans. J Leukoc Biol. (2001) 70:881–6. 10.1189/jlb.70.6.881 11739550

[37] Wenisch C Patruta S Daxböck F Krause R Hörl W. Effect of age on human neutrophil function. J Leukoc Biol. (2000) 67:40–5. 10.1002/jlb.67.1.40 10647996

[38] Lord JM Butcher S Killampali V Lascelles D Salmon M. Neutrophil ageing and immunesenescence. Mech Ageing Dev. (2001) 122:1521–35. 10.1016/S0047-6374(01)00285-8 11511394

[39] Fulop T Le Page A Fortin C Witkowski JM Dupuis G Larbi A. Cellular signaling in the aging immune system. Curr Opin Immunol. (2014) 29:105–11. 10.1016/j.coi.2014.05.007 24934647

[40] Fulop T Larbi A Douziech N Fortin C Guérard KP Lesur O. Signal transduction and functional changes in neutrophils with aging. Aging Cell. (2004) 3:217–26. 10.1111/j.1474-9728.2004.00110.x 15268755

[41] Fortin CF Lesur O Fulop T. Effects of aging on triggering receptor expressed on myeloid cells (TREM)-1-induced PMN functions. FEBS Lett. (2007) 581:1173–8. 10.1016/j.febslet.2007.02.029 17336301

[42] Guayerbas N Puerto M Víctor VM Miquel J De la Fuente M. Leukocyte function and life span in a murine model of premature immunosenescence. Exp Gerontol. (2002) 37:249–56. 10.1016/S0531-5565(01)00190-5 11772510

[43] Guayerbas N Catalán M Víctor VM Miquel J De la Fuente M. Relation of behaviour and macrophage function to life span in a murine model of premature immunosenescence. Behav Brain Res. (2002) 134:41–8. 10.1016/S0166-4328(01)00449-1 12191790

[44] Garrido A Cruces J Ceprián N Vara E de la Fuente M. Oxidative-inflammatory stress in immune cells from adult mice with premature aging. Int J Mol Sci. (2019) 20:769. 10.3390/ijms20030769 30759732 PMC6387005

[45] Garrido A Cruces J Ceprián N Hernández-Sánchez C De la Fuente M. Premature aging in behavior and immune functions in tyrosine hydroxylase haploinsufficient female mice. A longitudinal study. Brain Behav Immun. (2018) 69:440–55. 10.1016/j.bbi.2018.01.003 29341892

[46] da Costa RM Rodrigues D Pereira CA Silva JF Alves JV Lobato NS. Nrf2 as a potential mediator of cardiovascular risk in metabolic diseases. Front Pharmacol. (2019) 10:382. 10.3389/fphar.2019.00382 31031630 PMC6473049

[47] Viveros MP Arranz L Hernanz A Miquel J De la Fuente M. A model of premature aging in mice based on altered stress-related behavioral response and immunosenescence. Neuroimmunomodulation. (2007) 14:157–62. 10.1159/000110640 18073508

[48] Monti D Ostan R Borelli V Castellani G Franceschi C. Inflammaging and human longevity in the omics era. Mech Ageing Dev. (2017) 165:129–38. 10.1016/j.mad.2016.12.008 28038993

[49] Fülöp T Dupuis G Witkowski JM Larbi A. The role of immunosenescence in the development of age-related diseases. Rev Invest Clin. (2016) 68:84–91. 27103044

[50] Baylis D Bartlett DB Syddall HE Ntani G Gale CR Cooper C. Immune-endocrine biomarkers as predictors of frailty and mortality: a 10-year longitudinal study in community-dwelling older people. Age (Dordr). (2013) 35:963–71. 10.1007/s11357-012-9396-8 22388931 PMC3636387

[51] Bruunsgaard H Ladelund S Pedersen AN Schroll M Jørgensen T Pedersen BK. Predicting death from tumour necrosis factor-alpha and interleukin-6 in 80-year-old people. Clin Exp Immunol. (2003) 132:24–31. 10.1046/j.1365-2249.2003.02137.x 12653832 PMC1808682

[52] Harris TB Ferrucci L Tracy RP Corti MC Wacholder S Ettinger WH. Associations of elevated interleukin-6 and C-reactive protein levels with mortality in the elderly. Am J Med. (1999) 106:506–12. 10.1016/S0002-9343(99)00066-2 10335721

[53] Pawelec G. Hallmarks of human “immunosenescence”: adaptation or dysregulation? Immun Ageing. (2012) 9:15. 10.1186/1742-4933-9-15 22830639 PMC3416738

[54] Goronzy JJ Fang F Cavanagh MM Qi Q Weyand CM. Naive T cell maintenance and function in human aging. J Immunol. (2015) 194:4073–80. 10.4049/jimmunol.1500046 25888703 PMC4452284

[55] Di Benedetto S Derhovanessian E Steinhagen-Thiessen E Goldeck D Müller L Pawelec G. Impact of age, sex and CMV-infection on peripheral T cell phenotypes: results from the Berlin BASE-II Study. Biogerontology. (2015) 16:631–43. 10.1007/s10522-015-9563-2 25732234

[56] Olsson J Wikby A Johansson B Löfgren S Nilsson BO Ferguson FG. Age-related change in peripheral blood T-lymphocyte subpopulations and cytomegalovirus infection in the very old: the Swedish longitudinal OCTO immune study. Mech Ageing Dev. (2000) 121:187–201. 10.1016/S0047-6374(00)00210-4 11164473

[57] Strindhall J Skog M Ernerudh J Bengner M Löfgren S Matussek A. The inverted CD4/CD8 ratio and associated parameters in 66-year-old individuals: the Swedish HEXA immune study. Age (Dordr). (2013) 35:985–91. 10.1007/s11357-012-9400-3 22415616 PMC3636392

[58] Plunkett FJ Franzese O Finney HM Fletcher JM Belaramani LL Salmon M. The loss of telomerase activity in highly differentiated CD8+CD28–CD27– T cells is associated with decreased Akt (Ser473) phosphorylation. J Immunol. (2007) 178:7710–9. 10.4049/jimmunol.178.12.7710 17548608

[59] Larbi A Dupuis G Khalil A Douziech N Fortin C Fülöp T. Differential role of lipid rafts in the functions of CD4+ and CD8+ human T lymphocytes with aging. Cell Signal. (2006) 18:1017–30. 10.1016/j.cellsig.2005.08.016 16236485

[60] Taams LS Smith J Rustin MH Salmon M Poulter LW Akbar AN. Human anergic/suppressive CD4(+)CD25(+) T cells: a highly differentiated and apoptosis-prone population. Eur J Immunol. (2001) 31:1122–31. 10.1002/1521-4141(200104)31:4<1122::AID-IMMU1122>3.0.CO;2-P 11298337

[61] Martins PN Tullius SG Markmann JF. Immunosenescence and immune response in organ transplantation. Int Rev Immunol. (2014) 33:162–73. 10.3109/08830185.2013.829469 24127845 PMC5497513

[62] Seyda M Quante M Uehara H Slegtenhorst BR Elkhal A Tullius SG. Immunosenescence in renal transplantation: a changing balance of innate and adaptive immunity. Curr Opin Organ Transplant. (2015) 20:417–23. 10.1097/MOT.0000000000000210 26154914 PMC4819421

[63] Haynes L Lefebvre JS. Age-related deficiencies in antigen-specific CD4 T cell responses: lessons from mouse models. Aging Dis. (2011) 2:374–81. 22396889 PMC3295078

[64] Lang A Nikolich-Zugich J. Functional CD8 T cell memory responding to persistent latent infection is maintained for life. J Immunol. (2011) 187:3759–68. 10.4049/jimmunol.1100666 21890658 PMC4102748

[65] Sadighi Akha AA Miller RA. Signal transduction in the aging immune system. Curr Opin Immunol. (2005) 17:486–91. 10.1016/j.coi.2005.07.004 16061371

[66] Krogsgaard M Huppa JB Purbhoo MA Davis MM. Linking molecular and cellular events in T-cell activation and synapse formation. Semin Immunol. (2003) 15:307–15. 10.1016/j.smim.2003.09.002 15001169

[67] Decman V Laidlaw BJ Doering TA Leng J Ertl HC Goldstein DR. Defective CD8 T cell responses in aged mice are due to quantitative and qualitative changes in virus-specific precursors. J Immunol. (2012) 188:1933–41. 10.4049/jimmunol.1101098 22246631 PMC3320034

[68] Garcia GG Miller RA. Age-dependent defects in TCR-triggered cytoskeletal rearrangement in CD4+ T cells. J Immunol. (2002) 169:5021–7. 10.4049/jimmunol.169.9.5021 12391217

[69] Garcia GG Miller RA. Age-related defects in CD4+ T cell activation reversed by glycoprotein endopeptidase. Eur J Immunol. (2003) 33:3464–72. 10.1002/eji.200324310 14635057

[70] Haynes L Linton PJ Eaton SM Tonkonogy SL Swain SL. Interleukin 2, but not other common gamma chain-binding cytokines, can reverse the defect in generation of CD4 effector T cells from naive T cells of aged mice. J Exp Med. (1999) 190:1013–24. 10.1084/jem.190.7.1013 10510091 PMC2195647

[71] Wherry EJ Kurachi M. Molecular and cellular insights into T cell exhaustion. Nat Rev Immunol. (2015) 15:486–99. 10.1038/nri3862 26205583 PMC4889009

[72] Anderson AC Joller N Kuchroo VK. Lag-3, Tim-3, and TIGIT: co-inhibitory receptors with specialized functions in immune regulation. Immunity. (2016) 44:989–1004. 10.1016/j.immuni.2016.05.001 27192565 PMC4942846

[73] Parry RV Chemnitz JM Frauwirth KA Lanfranco AR Braunstein I Kobayashi SV. CTLA-4 and PD-1 receptors inhibit T-cell activation by distinct mechanisms. Mol Cell Biol. (2005) 25:9543–53. 10.1128/MCB.25.21.9543-9553.2005 16227604 PMC1265804

[74] Shimada Y Hayashi M Nagasaka Y Ohno-Iwashita Y Inomata M. Age-associated up-regulation of a negative co-stimulatory receptor PD-1 in mouse CD4+ T cells. Exp Gerontol. (2009) 44:517–22. 10.1016/j.exger.2009.05.003 19457448

[75] Channappanavar R Twardy BS Krishna P Suvas S. Advancing age leads to predominance of inhibitory receptor expressing CD4 T cells. Mech Ageing Dev. (2009) 130:709–12. 10.1016/j.mad.2009.08.006 19715717

[76] Lee KA Shin KS Kim GY Song YC Bae EA Kim IK. Characterization of age-associated exhausted CD8^+^ T cells defined by increased expression of Tim-3 and PD-1. Aging Cell. (2016) 15:291–300. 10.1111/acel.12435 26750587 PMC4783346

[77] Song Y Wang B Song R Hao Y Wang D Li Y. T-cell immunoglobulin and ITIM domain contributes to CD8. Aging Cell. (2018) 17:e12716. 10.1111/acel.12716 29349889 PMC5847879

[78] Johnston RJ Comps-Agrar L Hackney J Yu X Huseni M Yang Y. The immunoreceptor TIGIT regulates antitumor and antiviral CD8(+) T cell effector function. Cancer Cell. (2014) 26:923–37. 10.1016/j.ccell.2014.10.018 25465800

[79] Chew GM Fujita T Webb GM Burwitz BJ Wu HL Reed JS. TIGIT marks exhausted T Cells, correlates with disease progression, and serves as a target for immune restoration in HIV and SIV infection. PLoS Pathog. (2016) 12:e1005349. 10.1371/journal.ppat.1005349 26741490 PMC4704737

[80] Jiang N He J Weinstein JA Penland L Sasaki S He XS. Lineage structure of the human antibody repertoire in response to influenza vaccination. Sci Transl Med. (2013) 5:171ra19. 10.1126/scitranslmed.3004794 23390249 PMC3699344

[81] Gibson KL Wu YC Barnett Y Duggan O Vaughan R Kondeatis E. B-cell diversity decreases in old age and is correlated with poor health status. Aging Cell. (2009) 8:18–25. 10.1111/j.1474-9726.2008.00443.x 18986373 PMC2667647

[82] Frasca D Blomberg BB. Aging affects human B cell responses. J Clin Immunol. (2011) 31:430–5. 10.1007/s10875-010-9501-7 21318330 PMC5560853

[83] Stavnezer J Guikema JE Schrader CE. Mechanism and regulation of class switch recombination. Annu Rev Immunol. (2008) 26:261–92. 10.1146/annurev.immunol.26.021607.090248 18370922 PMC2707252

[84] Frasca D Landin AM Lechner SC Ryan JG Schwartz R Riley RL. Aging down-regulates the transcription factor E2A, activation-induced cytidine deaminase, and Ig class switch in human B cells. J Immunol. (2008) 180:5283–90. 10.4049/jimmunol.180.8.5283 18390709

[85] Frasca D Diaz A Romero M Landin AM Phillips M Lechner SC. Intrinsic defects in B cell response to seasonal influenza vaccination in elderly humans. Vaccine. (2010) 28:8077–84. 10.1016/j.vaccine.2010.10.023 20974306 PMC3223387

[86] Simell B Vuorela A Ekström N Palmu A Reunanen A Meri S. Aging reduces the functionality of anti-pneumococcal antibodies and the killing of Streptococcus pneumoniae by neutrophil phagocytosis. Vaccine. (2011) 29:1929–34. 10.1016/j.vaccine.2010.12.121 21236231

[87] Tortorella C Piazzolla G Spaccavento F Vella F Pace L Antonaci S. Regulatory role of extracellular matrix proteins in neutrophil respiratory burst during aging. Mech Ageing Dev. (2000) 119:69–82. 10.1016/S0047-6374(00)00171-8 11040403

[88] Schwarzenbach HR Nakagawa T Conroy MC de Weck AL. Skin reactivity, basophil degranulation and IgE levels in ageing. Clin Allergy. (1982) 12:465–73. 10.1111/j.1365-2222.1982.tb01645.x 6754133

[89] Smith P Dunne DW Fallon PG. Defective *in vivo* induction of functional type 2 cytokine responses in aged mice. Eur J Immunol. (2001) 31:1495–502. 10.1002/1521-4141(200105)31:5<1495::AID-IMMU1495>3.0.CO;2-8 11465106

[90] Nel HJ Hams E Saunders SP Mangan NE Smith P Atzberger A. Impaired basophil induction leads to an age-dependent innate defect in type 2 immunity during helminth infection in mice. J Immunol. (2011) 186:4631–9. 10.4049/jimmunol.1002995 21398616

[91] Mathur SK Schwantes EA Jarjour NN Busse WW. Age-related changes in eosinophil function in human subjects. Chest. (2008) 133:412–9. 10.1378/chest.07-2114 18252914 PMC2919352

[92] Ciaramella A Spalletta G Bizzoni F Salani F Caltagirone C Bossù P. Effect of age on surface molecules and cytokine expression in human dendritic cells. Cell Immunol. (2011) 269:82–9. 10.1016/j.cellimm.2011.04.010 21571262

[93] Fujihashi K Kiyono H. Mucosal immunosenescence: new developments and vaccines to control infectious diseases. Trends Immunol. (2009) 30:334–43. 10.1016/j.it.2009.04.004 19540811

[94] Gupta S. Role of dendritic cells in innate and adaptive immune response in human aging. Exp Gerontol. (2014) 54:47–52. 10.1016/j.exger.2013.12.009 24370374

[95] Huang C Wang Y Li X Ren L Zhao J Hu Y. Clinical features of patients infected with 2019 novel coronavirus in Wuhan, China. Lancet. (2020) 395:497–506. 10.1016/S0140-6736(20)30183-5 31986264 PMC7159299

[96] Jin Y Yang H Ji W Wu W Chen S Zhang W. Virology, epidemiology, pathogenesis, and control of COVID-19. Viruses. (2020) 12:372. 10.3390/v12040372 32230900 PMC7232198

[97] Xu Z Shi L Wang Y Zhang J Huang L Zhang C. Pathological findings of COVID-19 associated with acute respiratory distress syndrome. Lancet Respir Med. (2020) 8:420–2. 10.1016/S2213-2600(20)30076-X 32085846 PMC7164771

[98] Giamarellos-Bourboulis EJ Netea MG Rovina N Akinosoglou K Antoniadou A Antonakos N. Complex immune dysregulation in COVID-19 patients with severe respiratory failure. Cell Host Microbe. (2020) 27:992–1000.e3. 10.1016/j.chom.2020.04.009 32320677 PMC7172841

[99] Tai W He L Zhang X Pu J Voronin D Jiang S. Characterization of the receptor-binding domain (RBD) of 2019 novel coronavirus: implication for development of RBD protein as a viral attachment inhibitor and vaccine. Cell Mol Immunol. (2020) 17:613–20. 10.1038/s41423-020-0400-4 32203189 PMC7091888

[100] Hoffmann M Kleine-Weber H Schroeder S Krüger N Herrler T Erichsen S. SARS-CoV-2 cell entry depends on ACE2 and TMPRSS2 and is blocked by a clinically proven protease inhibitor. Cell. (2020) 181:271–80.e8. 10.1016/j.cell.2020.02.052 32142651 PMC7102627

[101] Canan CH Gokhale NS Carruthers B Lafuse WP Schlesinger LS Torrelles JB. Characterization of lung inflammation and its impact on macrophage function in aging. J Leukoc Biol. (2014) 96:473–80. 10.1189/jlb.4A0214-093RR 24935957 PMC4632167

[102] Velazquez-Salinas L Verdugo-Rodriguez A Rodriguez LL Borca MV. The role of interleukin 6 during viral infections. Front Microbiol. (2019) 10:1057. 10.3389/fmicb.2019.01057 31134045 PMC6524401

[103] Xiong Z Leme AS Ray P Shapiro SD Lee JS. CX3CR1+ lung mononuclear phagocytes spatially confined to the interstitium produce TNF-α and IL-6 and promote cigarette smoke-induced emphysema. J Immunol. (2011) 186:3206–14. 10.4049/jimmunol.1003221 21278339 PMC3912553

[104] Lagunas-Rangel FA Chávez-Valencia V. High IL-6/IFN-γ ratio could be associated with severe disease in COVID-19 patients. J Med Virol. (2020). 10.1002/jmv.25900. [Epub ahead of print]. 32297995 PMC7262117

[105] Wang F Hou H Luo Y Tang G Wu S Huang M. The laboratory tests and host immunity of COVID-19 patients with different severity of illness. JCI Insight. (2020) 5:e137799. 10.1172/jci.insight.137799 32324595 PMC7259533

[106] Han H Ma Q Li C Liu R Zhao L Wang W. Profiling serum cytokines in COVID-19 patients reveals IL-6 and IL-10 are disease severity predictors. Emerg Microbes Infect. (2020) 9:1123–30. 10.1080/22221751.2020.1770129 32475230 PMC7473317

[107] Zhang S Gan J Chen BG Zheng D Zhang JG Lin RH. Dynamics of peripheral immune cells and their HLA-G and receptor expressions in a patient suffering from critical COVID-19 pneumonia to convalescence. Clin Transl Immunology. (2020) 9:e1128. 10.1002/cti2.1128 32399213 PMC7211507

[108] Chen G Wu D Guo W Cao Y Huang D Wang H. Clinical and immunological features of severe and moderate coronavirus disease 2019. J Clin Invest. (2020) 130:2620–9. 10.1172/JCI137244 32217835 PMC7190990

[109] Day JW Fox TA Halsey R Carpenter B Kottaridis PD. IL-1 blockade with anakinra in acute leukaemia patients with severe COVID-19 pneumonia appears safe and may result in clinical improvement. Br J Haematol. (2020) 190:e80–3. 10.1111/bjh.16873 32438450 PMC7280623

[110] Pontali E Volpi S Antonucci G Castellaneta M Buzzi D Tricerri F. Safety and efficacy of early high-dose IV anakinra in severe COVID-19 lung disease. J Allergy Clin Immunol. (2020) 146:213–5. 10.1016/j.jaci.2020.05.002 32437739 PMC7211718

[111] Xu X Han M Li T Sun W Wang D Fu B. Effective treatment of severe COVID-19 patients with tocilizumab. Proc Natl Acad Sci U S A. (2020) 117:10970. 10.1073/pnas.2005615117 32350134 PMC7245089

[112] Camp JV Jonsson CB. A role for neutrophils in viral respiratory disease. Front Immunol. (2017) 8:550. 10.3389/fimmu.2017.00550 28553293 PMC5427094

[113] Zhang B Zhou X Zhu C Feng F Qiu Y Feng J. Immune phenotyping based on neutrophil-to-lymphocyte ratio and IgG predicts disease severity and outcome for patients with COVID-19. medRxiv. (2020). 10.1101/2020.03.12.20035048 32719810 PMC7350507

[114] Wen W Su W Tang H Le W Zhang X Zheng Y Immune cell profiling of COVID-19 patients in the recovery stageby single-cell sequencing. Cell Dis. (2020) 6:31 10.1038/s41421-020-0168-9 PMC7197635 32377375

[115] Wilk AJ Rustagi A Zhao NQ Roque J Martínez-Colón GJ McKechnie JL. A single-cell atlas of the peripheral immune response in patients with severe COVID-19. Nat Med. (2020) 26:1070–76. 10.1101/2020.04.17.20069930 32514174 PMC7382903

[116] Zheng M Gao Y Wang G Song G Liu S Sun D. Functional exhaustion of antiviral lymphocytes in COVID-19 patients. Cell Mol Immunol. (2020) 17:533–5. 10.1038/s41423-020-0402-2 32203188 PMC7091858

[117] Tam A Wadsworth S Dorscheid D Man SF Sin DD. The airway epithelium: more than just a structural barrier. Ther Adv Respir Dis. (2011) 5:255–73. 10.1177/1753465810396539 21372121

[118] Whitsett JA Alenghat T. Respiratory epithelial cells orchestrate pulmonary innate immunity. Nat Immunol. (2015) 16:27–35. 10.1038/ni.3045 25521682 PMC4318521

[119] Josset L Menachery VD Gralinski LE Agnihothram S Sova P Carter VS. Cell host response to infection with novel human coronavirus EMC predicts potential antivirals and important differences with SARS coronavirus. mBio. (2013) 4:e00165–13. 10.1128/mBio.00165-13 23631916 PMC3663187

[120] Brandenberger C Kling KM Vital M Christian M. The role of pulmonary and systemic immunosenescence in acute lung injury. Aging Dis. (2018) 9:553–65. 10.14336/AD.2017.0902 30090646 PMC6065297

[121] Kling KM Lopez-Rodriguez E Pfarrer C Mühlfeld C Brandenberger C. Aging exacerbates acute lung injury-induced changes of the air-blood barrier, lung function, and inflammation in the mouse. Am J Physiol Lung Cell Mol Physiol. (2017) 312:L1–L12. 10.1152/ajplung.00347.2016 27815259

[122] Schouten LR van Kaam AH Kohse F Veltkamp F Bos LD de Beer FM. Age-dependent differences in pulmonary host responses in ARDS: a prospective observational cohort study. Ann Intensive Care. (2019) 9:55. 10.1186/s13613-019-0529-4 31089908 PMC6517452

[123] Zhou F Yu T Du R Fan G Liu Y Liu Z. Clinical course and risk factors for mortality of adult inpatients with COVID-19 in Wuhan, China: a retrospective cohort study. Lancet. (2020) 395:1054–62. 10.1016/S0140-6736(20)30566-3 32171076 PMC7270627

[124] Thevarajan I Nguyen THO Koutsakos M Druce J Caly L van de Sandt CE. Breadth of concomitant immune responses prior to patient recovery: a case report of non-severe COVID-19. Nat Med. (2020) 26:453–5. 10.1038/s41591-020-0819-2 32284614 PMC7095036

[125] Liu W Fontanet A Zhang PH Zhan L Xin ZT Baril L. Two-year prospective study of the humoral immune response of patients with severe acute respiratory syndrome. J Infect Dis. (2006) 193:792–5. 10.1086/500469 16479513 PMC7109932

[126] Diao B Wang C Tan Y Chen X Liu Y Ning L. Reduction and functional exhaustion of T cells in patients with coronavirus disease 2019 (COVID-19). medRxiv. (2020) 11:827. 10.3389/fimmu.2020.00827 32425950 PMC7205903

[127] Nikolich-Zugich J. Ageing and life-long maintenance of T-cell subsets in the face of latent persistent infections. Nat Rev Immunol. (2008) 8:512–22. 10.1038/nri2318 18469829 PMC5573867

[128] Becklund BR Purton JF Ramsey C Favre S Vogt TK Martin CE. The aged lymphoid tissue environment fails to support naïve T cell homeostasis. Sci Rep. (2016) 6:30842. 10.1038/srep30842 27480406 PMC4969611

[129] Fessler J Ficjan A Duftner C Dejaco C. The impact of aging on regulatory T-cells. Front Immunol. (2013) 4:231. 10.3389/fimmu.2013.00231 23964277 PMC3734364

[130] Alpert A Pickman Y Leipold M Rosenberg-Hasson Y Ji X Gaujoux R. A clinically meaningful metric of immune age derived from high-dimensional longitudinal monitoring. Nat Med. (2019) 25:487–95. 10.1038/s41591-019-0381-y 30842675 PMC6686855

